# STAT3: An Anti-Invasive Factor in Colorectal Cancer?

**DOI:** 10.3390/cancers6031394

**Published:** 2014-07-03

**Authors:** Petrus Rudolf de Jong, Ji-Hun Mo, Alexandra R. Harris, Jongdae Lee, Eyal Raz

**Affiliations:** 1 Department of Medicine, University of California, San Diego, 9500 Gilman Dr. MC 0663, La Jolla, CA 92093, USA; E-Mails: pdejong@ucsd.edu (P.R.J.); arh5ru@virginia.edu (A.R.H.); eraz@ucsd.edu (E.R.); 2 Department of Otorhinolaryngology, Dankook University College of Medicine, 16-5 Anseo-dong, Cheonan, Chungcheongnam-do 330-715, Korea; E-Mail: jihunmo@gmail.com

**Keywords:** signal transducer and activator of transcription 3 (STAT3), colorectal cancer (CRC), metastasis, epithelial to mesenchymal transition (EMT), Snail-1 (SNAI-1), glycogen synthase kinase 3β (GSK3β), Snail, adenomatous polyposis coli (APC), tight junction (TJ), matrix metalloproteinases (MMPs)

## Abstract

Signal Transducer and Activator of Transcription 3 (STAT3) is activated in a majority of cancers, and promotes tumorigenesis and even metastasis through transcriptional activation of its target genes. Recently, we discovered that STAT3 suppresses epithelial-to-mesenchymal transition (EMT) and thus metastasis in a mouse model of colorectal cancer (CRC), while it did not affect the overall tumor burden. Furthermore, we found that STAT3 in intestinal epithelial cells (IEC) suppresses EMT by regulating stability of an EMT inducer, SNAI-1 (Snail-1). Here, STAT3 functions as an adaptor rather than a transcription factor in the post-translational modification of SNAI-1. In this review, we discuss the unexpected and contradictory role of STAT3 in metastasis of CRC and its clinical implications.

## 1. Colorectal Cancer (CRC)

CRC is the second most frequent cancer, after lung cancer, that leads to death in the US and Europe [1]. The development of sporadic human CRC is driven by the accumulation of somatic mutations in oncogenes (gain-of-function) and/or tumor-suppressor genes (loss-of-function), as well as epigenetic changes. Three etiological subtypes of genomic instability in CRC have been described, including chromosomal instability (CIN), microsatellite instability (MSI), and its related phenomenon known as the CpG island methylator phenotype (CIMP). 

CIN is the most common cause of CRC (80%–85%) and is associated with a poor prognosis [2]. CIN in CRC is characterized by chromosomal rearrangements (unbalanced translocation, deletion) and abnormality in chromosome numbers (gain or loss of whole chromosomes) [3]. Additionally, CIN is caused by somatic mutations in genes that regulate the mitotic spindle checkpoint, DNA replication checkpoints, DNA damage checkpoints, chromosome metabolism, or centrosome function [2,4]. The CIN phenotype is used to measure intercellular heterogeneity in chromosome number and describe cancers with aneuploidy, polyploidy, or loss of heterozygosity (LOH) [5]. Gain or loss of the chromosomal copy number contributes to development and/or progression of cancer via inactivation of tumor suppressor genes, activation of oncogenes, or change in gene dosage (increasing or decreasing expression of these genes) [6]. In fact, copy number alterations seem to be the major mechanism for transcriptional deregulation of cancer genes in CRC [7]. 

Loss of function of mismatch-repair (MMR) genes (MLH1, MSH2, MSH6, and PMS2) is responsible for the epiphenomenon of MSI, which is associated with mutations in tumor-suppressor genes. CIMP involves epigenetic silencing of tumor suppressor genes, such as MLH1, due to aberrant DNA methylation (promoter hypomethylation of oncogenes or hypermethylation of tumor suppressor genes). This oncogenic mechanism shows overlap with MSI in CRC subgroups [8]. MSI occurs in ~15% of CRC patients, and MSI^+^ CRC has a better prognosis when compared with CIN^−^ or MSI^− ^CRC. Approximately 20% of CRC patients are CIMP^+^.

Whereas MSI^+^ CIMP^+^ CRC confers a better prognosis for CRC, patients with MSI^−^ CIMP^+^ CRC have a worse prognosis [9]. Recently, De Sousa e Melo *et al.* [10] confirmed the existence of multiple CRC subtypes by gene expression profiling. They identified three categories, including colon cancer subtypes that correspond to the CIN^+^ or MSI/CIMP^+^ class, respectively. In addition, they identified a third subclass that was characterized by hallmarks of epithelial-mesenchymal transition (EMT), shared features with sessile serrated adenoma, and conferred a poor prognosis in CRC [10]. Sadanandam *et al.* proposed an alternative classification system based on gene expression profiling of primary CRC tumors [11]. These novel CRC subtypes included enterocyte, goblet-like, transit-amplifying, inflammatory, and stem-like tumor cells, correlating with their colon-crypt location, Wnt activity, and response to adjuvant or metastatic treatment [11]. In addition to their prognostic features, a higher resolution of CRC subtypes allows for better stratification of CRC patients in clinical trials, and may better predict their response to targeted therapy. Importantly, despite all these novel insights, the molecular events that lead to tumor invasion and metastasis are still largely unknown.

With regard to oncogenic pathways, CIN is associated with inactivating mutations in the *APC* (adenomatous polyposis coli) gene or, to a lesser extent, gain-of-function mutations in the *CTNNB1* (β-catenin) gene. These mutations result in the formation of an early adenoma. This is followed by the adenoma-to-carcinoma transition (in CIN) through sequential genetic mutations in oncogenes (e.g., *KRAS*, *PIK3CA*, *PTEN*, or *EGFR/ERBB* family members) and/or tumor suppressor genes (e.g., *TP53*, *SMAD4*, *BAX*) [12,13]. While germline mutations in the *APC* gene are responsible for familial adenomatous polyposis (FAP), somatic mutations in *APC* occur in ~90% of sporadic colorectal tumors. These mutations in *APC* lead to unrestricted activation of β-catenin, which in turn activates many genes responsible for tumorigenesis, such as *MYC*. In fact, an integrated genetic analysis by The Cancer Genome Atlas (TCGA) Network showed that nearly 100% of CRC tumors showed changes in transcriptional targets of MYC [13]. 

Most of CRC-related mortality is due to metastasis, which develops in at least 50% of CRC patients, and the majority of metastatic tumors are not surgically resectable [14]. Thus, it is critical to understand the mechanisms underlying CRC metastasis.

The most widely used animal model of CRC is the *Apc^min/+^* mouse strain in which multiple intestinal neoplasia (min) spontaneously develop, mostly in the small intestine. Adenomas in this model generally do not progress to adenocarcinomas. Nonetheless, this model has been an important tool to understand tumorigenesis and metastasis of CRC. We made a conditional deletion of STAT3 in IEC of these mice (*Apc^min^*^/+^/*Stat3^IEC-KO^* mice) to investigate the role of STAT3 in CRC and found an anti-carcinogenic role for STAT3 [15], which was unexpected given a plethora of literature supporting the pro-oncogenic role of STAT3. Musteanu *et al.* also reported this anti-invasive role for STAT3 [16]. Together, these data suggest that STAT3 suppresses malignant transformation of adenomas in the *Apc^min/+^* mouse strain.

## 2. STAT3 Signaling and Its Role in Cancers

STAT3 plays an essential role in a variety of physiological functions, including development, proliferation, and immune defense [17,18,19,20]. STAT3 is phosphorylated on a tyrosine residue (Tyr-705) by an upstream kinase, JAK2 (Janus kinase 2). In addition to phosphorylation at Tyr-705, STAT3 is phosphorylated at Ser-727 and acetylated at Lys-685 [21], which is thought to be required for its full transcriptional activity. The phosphorylated protein forms either a homodimer or a heterodimer with other STAT proteins, then translocates to the nucleus and transcribes target genes. However, STAT3 can also regulate expression of certain target genes (e.g., RANTES, IL-6, IL-8, Met, and M-Ras) in a phosphorylation-independent manner [22,23]. Several non-transcriptional functions of STAT3 have also been reported. STAT3 functions as an adaptor protein, connecting IFNAR1 (interferon α receptor 1) and the p85 regulatory subunit of PI3K (phosphoinositide 3-kinase) [24], and it inhibits stathmin that depolymerizes microtubules [25]. Activated STAT3, through interaction with phosphorylated paxillin and focal adhesion kinase (FAK), was shown to promote ovarian cancer cell invasiveness [26].

Numerous *in vitro* and *in vivo* studies indicate that STAT3 promotes tumorigenesis of a variety of cancers, thus it is generally recognized as an oncogene. STAT3 is activated in 70% of all solid and hematological tumors [27,28], and it was found activated in 72% of colorectal carcinomas but only in 18% of colorectal adenomas [29]. Furthermore, p-STAT3 expression is associated with a poor prognosis in CRC, independent of MSI, CIMP, BRAF, or KRAS mutation status [30]. STAT3 is rarely mutated in cancer cells but rather activated by upstream signals, such as receptor tyrosine kinses (RTKs), mutated JAKs, or oncogenic cellular tyrosine kinases (CTKs), such as Src. Recently, somatic mutations in the SH2 dimerization and activation domain of STAT3 were discovered in 40% of patients with large granular lymphocytic leukemia, and these mutations were associated with enhanced phosphorylation of STAT3 and its localization in the nucleus [31]. 

## 3. STAT3 in Intestinal Epithelial Cells (IEC) Promotes Cell Survival and Epithelial Barrier Function

**Figure 1:**
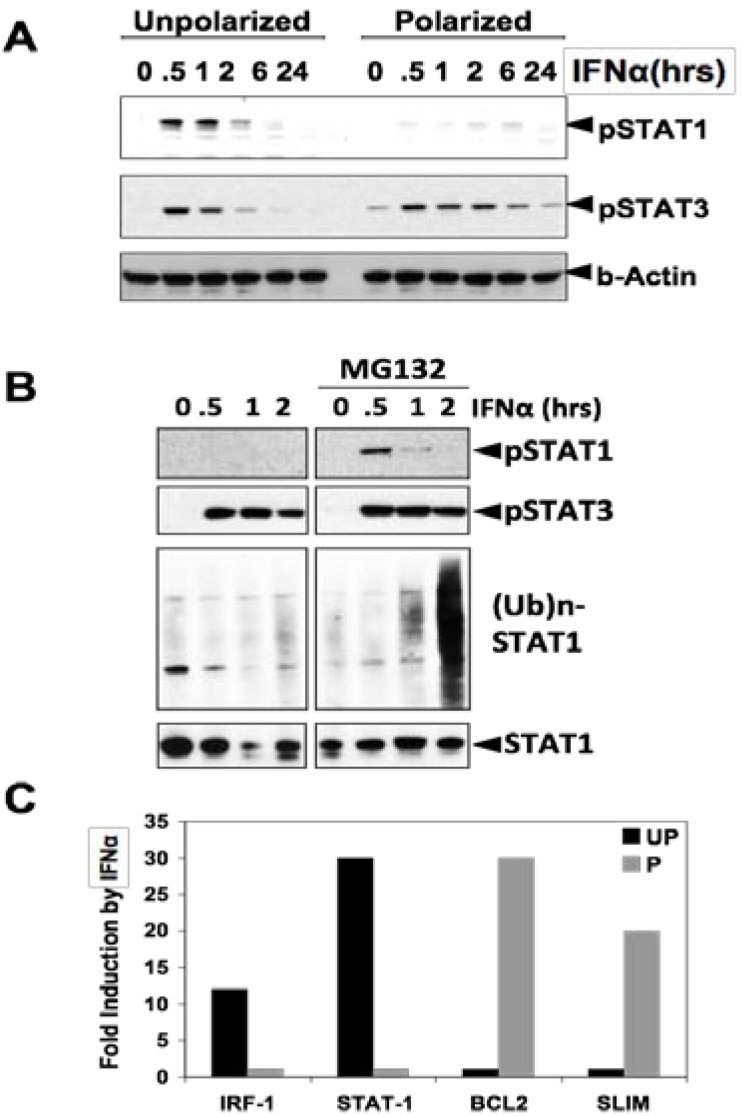
STAT3 is preferentially activated by IFNα, while STAT1 is degraded by the proteasome in polarized IEC. (**A**) Polarized or unpolarized HCA-7 cells were stimulated with IFNα (10 ng/mL) for the indicated time period and the indicated proteins were measured by immunoblotting; (**B**) Polarized HCA-7 cells were stimulated with IFNα(10 ng/mL) for the indicated time period in the absence or presence of MG132 (10 µM) and the indicated proteins were measured by immunoblotting. To detect ubiquitination of STAT1, STAT1 was immunoprecipitated and blotted with anti-ubiquitin antibody; (**C**) Polarity-dependent gene activation by IFNα in IECs. Polarized (P) or unpolarized (UP) HCA-7 cells were stimulated with IFNα (10 ng/mL) for 1 hr and the induction of indicated genes by IFNα was measured by qPCR. The data represents one of two independent experiments with similar results.

Membranes of IEC, like any epithelium, are polarized with tight junctions as the boundary. The apical membrane, facing the lumen, expresses distinctive proteins (e.g., digestive enzymes, nutrient transporters, *etc*.) in IEC. Conversely, proteins in the basolateral membrane resemble those in other unpolarized cell types [32]. This is achieved by complex and coordinated protein sorting and targeting machinery [33]. We have shown that TLR9 is expressed in both the apical and basolateral membranes, but only apical TLR9 generates a response that suppresses the inflammatory responses of basolateral TLRs, including TLR3, TLR5, and TLR9 [32]. This was surprising for three main reasons. First, since TLR9 is expressed in endosomes of immune cells, our data indicate that localization of TLR9 is not determined by its primary sequence. Second, it indicates that different downstream effectors of the same receptor (TLR9) are utilized depending on the location of the receptor. Whether this is unique to TLR9 remains to be seen. Third, when IEC lose polarity (by culturing on a plastic surface), TLR9 signaling mainly resembles that of the basolateral TLR9 (inflammatory). We suspect that intracellular signaling cascades in IEC could be altered under pathological conditions where polarity is disrupted (e.g., inflammation or cancer). Recently, we found such an example: interferon α (IFNα) activates STAT3 preferentially in polarized IEC, while it activates both STAT1 and STAT3 to the same extent in unpolarized IEC (Figure 1A). STAT1 is rapidly degraded by the proteasome upon IFNα stimulation in polarized IEC, which can be rescued by blockade of the proteasome (Figure 1B). Regulation of STAT1 signaling by polarization in IEC resulted in a distinctive pattern of gene expression upon IFNα stimulation (Figure 1C): more inflammatory genes, such as IRF-1 and STAT1, were induced in unpolarized IEC, whereas anti-apoptotic genes, such as Bcl-2, were induced in polarized IEC. SLIM, a ubiquitin ligase of STAT1 [34], was also induced by IFNα in polarized IEC, which is consistent with the result that STAT1 is degraded by the proteasome under this condition. Thus, from these results and other evidence in the literature, it was expected that STAT3 in IEC should promote tumorigenesis by preventing apoptosis. 

**Figure 2:**
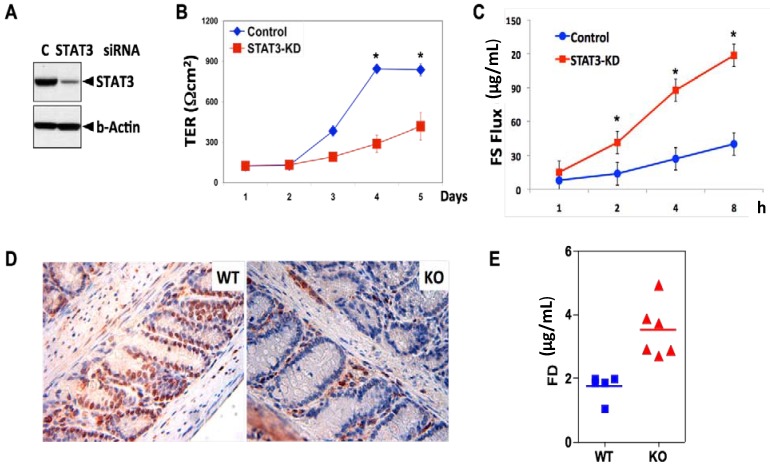
STAT3 promotes polarization and barrier function of IEC *in vitro* and *in vivo*. (**A**) STAT3 in HCA-7 cells was silenced by RNAi and the indicated proteins were measured by immunoblotting; (**B**) Cells transfected with the indicated siRNA were plated on a Transwell plate and transepithelial electrical resistance (TER) was measured at the indicated time. (*n* = 6) (* represents *p* value < 0.05, ANOVA); (**C**) The barrier function of HCA-7 cells was assessed by using FITC-sulfonic acid (FS). FS was measured five days after transfection (*n* = 6); (**D**) The deletion of STAT3^IEC^ in mice was confirmed by immunohistochemistry (IHC) as described [37]. STAT3 staining (brown) in the KO mice is confined to mononuclear cells in the LP (Lamina Propria) but is absent in IEC; (**E**) Intestinal permeability of KO mice is significantly higher than that of WT mice (*p* = 0.001). Mice were gavaged with FITC-dextran (FD, MW 4000, 6 mg/mouse) and the level of FD in serum was measured 90 min post administration.

However, we also found that STAT3 promotes polarization of IEC, measured by trans-epithelial electrical resistance (TER), and reduces trans-epithelial permeability, a measure of barrier function (Figure 2). Since carcinomas typically lose polarity before metastasis, our data also suggest that STAT3 might inhibit EMT and thus metastasis of CRC.

## 4. STAT3 Does Not Affect Intestinal Tumor Initiation

Contrary to the expectation that STAT3 would be a major driver of tumorigenesis but consistent with the data by Musteanu *et al.* [16], we found that STAT3 in IEC plays only a minor role in tumor initiation in the *Apc^min/+^* model. However, both groups observed a dramatic increase in tumor invasion of *Apc^min/+^* tumors in the intestines upon deletion of *Stat3* in IEC *in vivo*. It must be noted that some of our observations in the *Apc^min^*^/+^/*Stat3^IEC-KO^* strain differ from those reported by Musteanu *et al* [16]*.* Whereas they observed a slight reduction in tumor multiplicity at a late stage [15], we found that the number of adenomas in the small and large intestine did not significantly change in *Apc^min^*^/+^/*Stat3^IEC-KO^* mice [15]. Furthermore, they observed an increased rate of IEC proliferation and nuclear translocation of β-catenin in tumors of *Apc^min^*^/+^/*Stat3^IEC-KO^* mice [16], whereas we found no differences in these parameters [15]. Interestingly, Musteanu *et al.* [16] observed increased p-STAT3 levels in *Apc^min/+^* adenomas [16], and we found that total STAT3 expression in adenomas was substantially higher than that in normal IEC (unpublished data). These data both suggest that STAT3 is highly activated in tumor cells, since STAT3 activates its own transcription [35]. In the *Apc^min/+^* model, β-catenin is the driver of tumorigenesis and STAT3 has been directly implicated in the activation of β-catenin. For example, the expression of a dominant negative (DN)-STAT3 or pharmacological inhibition of JAK/STAT3 signaling induced translocation of β-catenin from the nucleus to the cytoplasm. This resulted in reduced transcriptional activity of β-catenin and inhibition of cell proliferation [36]. In contrast, Musteanu *et al.* proposed that STAT3^IEC-KO^ resulted in down-regulation of the cell adhesion protein, CEACAM1, with a concomitant increase in nuclear β-catenin [16]. However, we did not observe this change in localization of β-catenin and therefore propose an alternative model in which STAT3 negatively regulates EMT. 

It is important to note that STAT3 is essential for tumorigenesis in the colitis-associated cancer (CAC) model in mice [38,39]. This model employs a carcinogen (azoxymethane) to induce mutagenesis, which is followed by repeated administration of DSS (dextran sulfate sodium) to induce chronic colitis [40]. Metastasis in this model is still dependent on mutations in the β-catenin gene [41]. Although dysfunctional APC can affect molecules other than β-catenin, β-catenin is essential for intestinal tumorigenesis. How does STAT3 play such a contradictory role in sporadic versus colitis-induced CRC? We found that STAT3-deficient IEC are highly sensitive to DSS-induced death (unpublished data in *Stat3^IEC-KO^* mice), as reported by Grivennikov *et al.* [38]. It is possible that potential STAT3-deficient cancer cells in this model may not have survived the repeated DSS treatments in the CAC model. The *Apc^min/+^* model is not driven by the carcinogenic effects of inflammation or any other chemical interventions. Thus, the effect of epithelial STAT3 signaling on tumor initiation is likely very different in sporadic CRC versus CAC. 

## 5. STAT3 Suppresses Adenoma to Adenocarcinoma Transition

While tumorigenesis was only minimally affected by deletion of STAT3, tumors in *Apc^min^*^/+^/*Stat3^IEC-KO^* mice invaded deeply into the mucosal stroma, submucosa, and muscle, indicating that STAT3 suppresses metastasis [15,16]. Epithelial-to-mesenchymal transition (EMT) is an integral process of metastasis and generally considered a prerequisite [42,43,44,45]. Epithelial cells are tightly interlocked with each other by junctional complexes, including tight junctions (TJ) and adherens junctions (Figure 1). Loss of junctional proteins such as E-cadherin, an adherens junction protein, is a hallmark of EMT and metastasis in many cancers [46,47,48]. Cancer cells that acquired a mesenchymal phenotype express prototypical mesenchymal proteins, such as vimentin and fibronectin, as well as various metalloproteinases. STAT3 is reportedly involved in IL-6-induced EMT in head and neck tumor metastasis [49], and promotes metastasis of melanoma to the brain by induction of the extracellular matrix-degrading metalloproteinases, including MMP-2 and MMP-9 [50]. 

In contrast, we found that STAT3 plays an opposite role in the *Apc^min^*^/+^ model. Intestinal adenomas in *Apc^min^*^/+^ mice did not show down-regulation of E-cadherin, while there was a remarkable down-regulation of the TJ protein, CLDN-3 (claudin-3) when compared to normal IEC [51]. Furthermore, expression of CLDN-3 and CLDN-5 in tumor cells was markedly decreased in tumor cells of *Apc^min^*^/+^/*Stat3^IEC-KO^* mice compared with those in *Apc^min^*^/+^ mice; there was no difference in E-cadherin or occludin levels. More importantly, all tumors in *Apc^min^*^/+^/*Stat3^IEC-KO^* mice expressed high levels of vimentin and, in some tumors, fibronectin, whereas those in *Apc^min^*^/+^ mice rarely expressed either [15]. Results showing the loss of TJ proteins [51] and slight elevation of vimentin [52] suggest that a low grade of EMT is initiated in *Apc^min^*^/+^ mice and indicate that STAT3 deletion can accelerate this process. As discussed above, Musteanu *et al.* proposed that STAT3 impairs invasion of IEC tumors via the cell adhesion molecule, CEACAM1 [16]. Therefore, STAT3 appears to be intimately involved in cell adhesion of IEC and other epithelial cell types.

Expression of activated STAT3 in colon cancer cells (HT-29 or CoGa-1) induced metalloproteinases MMP1, MMP3, MMP7, and MMP9, which were demonstrated to aid invasiveness of these cells [53]. Our data showed that, while MMP9 and MMP15 (MT2-MMP) expression in tumor cells was negligible in both *Apc^min^*^/+^ and *Apc^min^*^/+^/*Stat3^IEC-KO^* mice, MMP7 expression in tumor cells was significantly lower in *Apc^min^*^/+^/*Stat3^IEC-KO^* mice. These data indicate that MMP7, MMP9, and MMP15 are not likely to contribute to tumor invasion in these mice. However, MMP14 (MT1-MMP) expression in tumor cells was highly elevated in *Apc^min^*^/+^/*Stat3^IEC-KO^* mice but undetectable in *Apc^min^*^/+^ mice. Since MMP14 is particularly efficient in hydrolyzing basement membranes [54,55], it may be involved in tumor invasion in *Apc^min^*^/+^/*Stat3^IEC-KO^* mice. Furthermore, overexpression of the *MT1-MMP* gene is reported as a useful predictor of outcomes in patients with CRC [56]. Several MT1-MMP inhibitors are under development for cancer therapy [57]. 

## 6. STAT3 Inhibits Tumor Invasion via Regulation of an EMT Inducer SNAI-1

EMT is induced by a group of transcription repressors, including SNAI-1, Slug, Zeb-1, Zeb-2, Twist, E47, and KLF. These factors directly or indirectly repress transcription of E-cadherin and other junction proteins, including claudins and desmosomes, thereby facilitating EMT [42,43,44,45]. Among the tested EMT inducers, only SNAI-1 was significantly elevated in tumors of *Apc^min^*^/+^/*Stat3^IEC-KO^* mice [15]. Silencing STAT3 in a few human CRC cell lines also induced expression of SNAI-1 but not other EMT inducers, while overexpression of STAT3-WT suppressed SNAI-1 expression. STAT3 knockdown significantly increased the invasiveness of a CRC cell line, HCT116, which was completely dependent on SNAI-1 [15]. Our data are directly contradictory to a previous study in which STAT3 reportedly increased the invasiveness of HT-29 or CoGa-1 [53]. HCT116 cells are *APC^WT^*, whereas both HT-29 and CoGa-1 cell lines carry *APC* mutations. This suggests that the suppression of EMT by STAT3 is not dependent on constitutive activation of Wnt signaling. Furthermore, Xiong *et al.* reported that STAT3 mediates down-regulation of E-cadherin through Zeb-1, thereby promoting EMT and the invasive properties of CRC [58], which is also contradictory to our findings. Their observations were mainly done in human CRC lines SW1116 and LoVo [58], which were derived from a primary tumor and metastasized CRC, respectively, and carry *APC* mutations. Finally, another report suggested that STAT3 inhibition negatively regulated the migratory and invasive properties of HCT116 cells [59]. Differential *in vitro* conditions may cause STAT3 to have opposite effects on EMT, even within the same cell line, an issue that remains to be clarified. The tumor microenvironment, which is lost in CRC cell line cultures, dictates the metastatic capacity of disseminated CRC cells *in vivo*. In this regard, the GP130/STAT3 signaling axis has been shown to contribute to CRC metastasis [60]. Thus, STAT3 displays both pro- and anti-metastatic effects in CRC, the outcome of which may be determined by the tumor microenvironment. 

## 7. STAT3 Coordinates SNAI-1 Stability as a Molecular Adaptor

**Figure 3:**
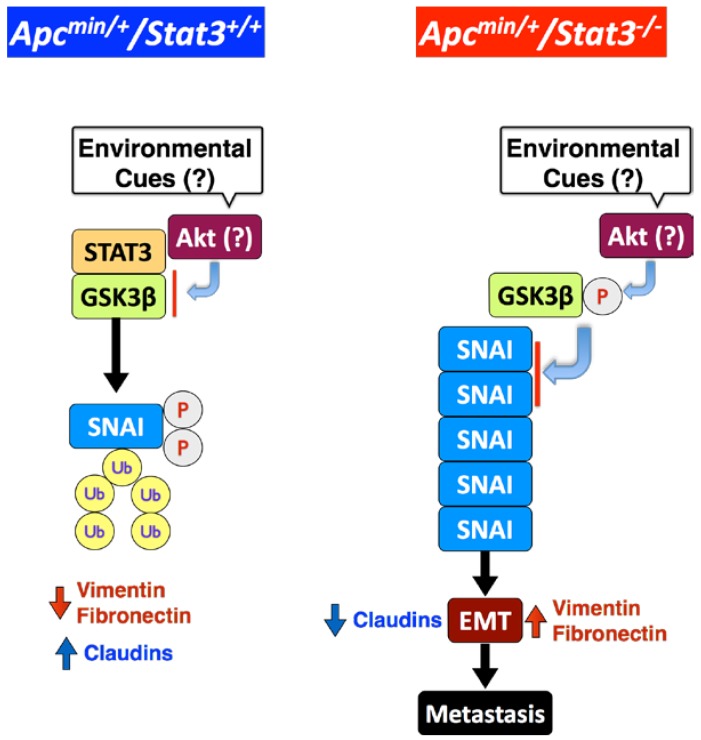
STAT3 suppresses EMT by promoting GSK3β-mediated phosphorylation and proteasomal degradation of SNAI-1 in IEC. STAT3 inhibits phosphorylation of GSK3β (active), which phosphorylates SNAI-1, leading to ubiquitination and degradation. Consequently, epithelial cell markers, such as claudins, are upregulated and mesenchymal markers, such as vimentin and fibronectin, are down-regulated. When STAT3 is deleted from IEC, unknown environmental cue(s) induce phosphorylation of GSK3β (inactive), most likely via the Akt pathway, leading to accumulation of SNAI and EMT.

Unexpectedly, STAT3 deletion or knockdown in IEC did not significantly affect the transcription of SNAI [15], indicating that STAT3 regulates SNAI-1 at a post-transcriptional level. SNAI in a CRC cell line was constitutively ubiquitinated and degraded by proteasomes. STAT3 knockdown increased the level of SNAI-1 but diminished the level of SNAI ubiquitination [15], indicating that STAT3 facilitates ubiquitination of SNAI-1. Phosphorylation of SNAI-1 by GSK3β at two different sites regulates the fate of SNAI-1 during EMT: phosphorylation of the first motif induces ubiquitination of SNAI-1, whereas phosphorylation of the second motif controls its subcellular localization [61]. As expected, we found that pharmacological inhibition or knockdown of GSK3β elevated the SNAI-1 level in a CRC cell line [15]. STAT3 and GSK3β co-immunoprecipitated, but expression of STAT3 protein was not affected by GSK3β or vise versa. However, whether STAT3 has to be phosphorylated to interact with GSK3β is still to be determined. STAT3 knockdown elevated phosphorylation on Ser9 of GSK3β, which inhibits its kinase activity [62]. GSK3β is also involved in activation of β-catenin, but GSK3α can compensate for the absence of GSK3β [63]. This may explain why there were no changes in β-catenin levels. Ectopic expression of a constitutively active GSK3β mutant in a CRC cell line not only reversed the SNAI-1 induction but also abrogated the invasiveness caused by STAT3 depletion [15]. In summary, these results demonstrate that STAT3 acts as a molecular adaptor to regulate GSK3β activity, and thus SNAI-1 expression and EMT (Figure 3). It is not clear how STAT3 regulates GSK3β activity, but it may be by acting as a bridge between GSK3β and SNAI-1 to facilitate phosphorylation. In addition, whether the interaction among these three proteins occurs in the cytoplasm or nucleus needs to be investigated. 

## 8. Clinical Implications

Because of the vast amount of literature supporting the oncogenic role of STAT3, several approaches to inhibit STAT3 activity have been developed for potential clinical trials. These include inhibitors of upstream molecules, such as IL-6 [64] or JAK2 [65,66], and direct inhibition of STAT3 with a STAT3 decoy oligonucleotide [67] or siRNA [68]. While the outcome of these clinical trials should tell us whether STAT3 inhibition is truly efficacious, caution is warranted for CRC trials with direct STAT3 inhibition. 

If our model is correct, we should expect adverse effects of direct inhibition of STAT3 in CRC, while targeting upstream molecules such as IL-6 or JAK2 is more complicated, as many targets other than STAT3 could be influenced by these approaches. Another possibility is that the outcome of direct inhibition of STAT3 in CRC is dependent on the underlying genetic or epigenetic background. This becomes increasingly complex when considering the role of STAT3 in non-tumor cells in metastasis (*i.e.*, the tumor microenvironment), as cancer drugs rarely discriminate normal cells from tumor cells. Only further investigations and the outcomes of clinical trials may provide more clarity. 

## 9. Conclusions

Our data and the data from Mesteanu *et al.* suggest that STAT3 can act as an anti-invasive factor under certain conditions [15,16]. The potential anti-EMT and anti-invasive role of STAT3 warrants rigorous follow-up studies, considering the depth of literature supporting its role as a strong promoter of tumorigenesis and eventual metastasis. Ongoing and future clinical trials on CRC with an agent directly inhibiting STAT3 activity should provide more insight into this paradoxical function of STAT3. 
